# Food for thought: more explicit guidance for inclusion of caregiver perspectives in health technology assessment

**DOI:** 10.1017/S0266462324004690

**Published:** 2024-12-12

**Authors:** Siobhan Bourke, Chris Skedgel, Yasmina Martí-Gil, Peter J. Neumann, Louis P. Garrison, Samantha Benham-Hermetz, Frauke Becker, Maria João Garcia

**Affiliations:** 1 Putnam, Patient Reported Outcomes, Ashby-De-La-Zouch, UK; 2 Office of Health Economics, London, UK; 3 F Hoffmann-La Roche AG, Basel, Switzerland; 4 Tufts Medical Center, Boston, MA, USA; 5 University of Washington - Seattle Campus, Comparative Health Outcomes, Policy, & Economics (CHOICE) Institute, Seattle, WA, USA; 6 Alzheimer’s Research UK, Cambridge, UK

**Keywords:** caregivers, carers, informal care, health technology assessment, methodological guidance

## Abstract

Caregivers can play an important role in supporting and caring for people with progressive, life-threatening, or debilitating conditions. However, this supportive role can expose caregivers to various detrimental financial, physical, and psychosocial issues. When evaluating medical technologies for reimbursement decisions, health technology assessment (HTA) agencies typically focus on the treatment’s impact on patients and ignore or downplay the impact on caregivers. Including caregiver impacts within a wider societal perspective may better enable health systems to maximize health benefits from available resources. However, the lack of clear guidance or methodological recommendations from decision makers on the inclusion of caregiver impacts limits the number of HTA submissions that consider these effects. We outline a conceptual framework based on intensity and duration of caregiving to guide researchers, industry, and decision makers when developing policies for the inclusion of caregiver outcomes and justify their inclusion based on expected caregiver burden in identified circumstances.

## Introduction

Health technologies can significantly impact the well-being of not just patients but also those around them, particularly informal caregivers. These caregivers, who provide unpaid care and support to family members, partners, or friends because of their health or social needs (including disability, frailty, or illness) (1), may see a reduction in caregiver burden as a result of treatment. These impacts can affect both informal caregivers’ and family members’ well-being, commonly known as “family spillover effects” (2), and can be measured by methodologies frequently used in health technology assessments (HTAs) (e.g., health-related quality of life (HRQoL) instruments (3) and/or the financial (direct/indirect) cost implications associated with caregiving) (4).

The current scope of international HTA methods used for informing reimbursement decisions and drug coverage policies, however, does not consistently or adequately consider these broader societal impacts of treatment, especially in the base case analysis (Table 1). HTA bodies, including the National Institute for Health and Care Excellence (NICE) in England, promote the inclusion of “all direct health effects for patients or, when relevant, carers” in its methods guide (5). Although NICE acknowledges the value of health benefits for caregivers, the inclusion of caregiver perspectives in HTA submissions remains relatively uncommon (14). This trend may reflect a lack of clear commitment from HTA bodies to consider the full scale and scope of the impact of new technologies on caregivers, potentially undervaluing interventions that may improve the delivery of care and therefore reduce the strain or burden experienced by caregivers (14). This lack of consideration could also discourage investment in additional caregiving-related research.

**Table 1.**
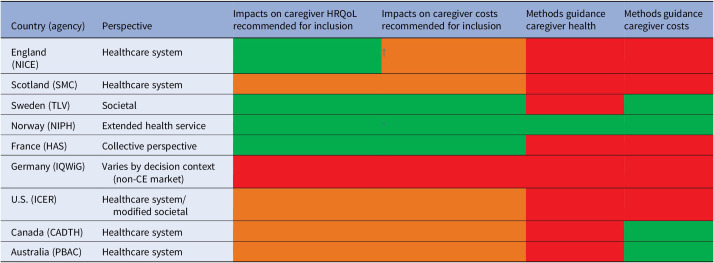
Overview of caregiver inclusion and methodology in HTA

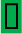
 recommended for inclusion/guidance available

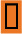
 recommended in supplementary analysis

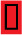
 no recommendation/guidance available

† Cost of the time providing informal care by family members, friends, or a partner that might otherwise have been provided by the NHS or personal social services (PSSs) may be considered outside of the base case.

* Includes recommendations for the estimation of cost for caregiving time for duration of treatment administration and/or travel time if different.

Abbreviations: NICE, National Institute for Health and Care Excellence (5); SMC, Scottish Medicines Consortium (6); TLV, Tandvårds- och läkemedelsförmånsverket (7); NIPH, Norwegian Institute of Public Health (8); HAS, Haute Autorité de santé (9); IQWiG, Institut für Qualität und Wirtschaftlichkeit im Gesundheitswesen (10); ICER, Institute for Clinical and Economic Review (11); CADTH, Canadian Agency for Drugs and Technologies in Health (12); PBAC, Pharmaceutical Benefits Advisory Committee (13); CEA, cost-effectiveness analysis.

HTA agencies worldwide take different approaches to considering caregiver perspectives formally. For example, the United Kingdom (UK), Sweden, France, and the Netherlands consider caregiver impacts in the cost per quality-adjusted life-year (QALY) framework, whereas other HTA agencies allow for the inclusion of qualitative evidence outside of the QALY framework (e.g., Germany and the U.S.) (15). The objective of HTA agencies operating under the QALY framework is to maximize the (quality-adjusted) quantity of health produced from the expenditure of a fixed healthcare budget (16). However, within a QALY framework, the justification of *whose* health should be maximized is unclear, that is, patient-only or also that of caregivers or wider “family spillover” effects. Promising steps have already been taken to include treatment benefits beyond patients’ HRQoL when evaluating new health technologies, going beyond health as the final outcome and including a new well-being measure (17) as well as a growing recognition that caregiver time is a valuable (and scarce) healthcare resource that should be considered as carefully as any other healthcare resource (1).

There is, however, an acknowledgment by some HTA agencies and leading academics that providing clearer guidance on the relevance and inclusion of a caregiver perspective in HTA could lead to a more consistent approach and produce higher-quality evidence (4). Many of the current HTA guidelines leave it to the submitting party to judge whether it is appropriate to include the caregiver perspective in submissions and offer little guidance – or incentive to do so. Although much of the existing literature has focused on *why* and *how* to consider spillovers, such as carer effects (4;18;19), we seek to address *when* such effects should be considered. This commentary article will introduce a conceptual framework designed to serve as a foundation for developing policies that include caregiver burden in identified disease areas and circumstances, aiming to assist decision makers, industry, and researchers in HTA.

## Considerations for future developments in HTA

**Figure 1.**
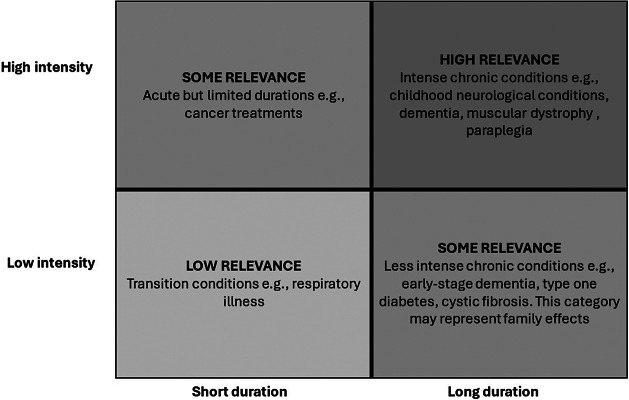
Carer burden framework.

Caregiving is a complex, multidimensional activity that can impact a wide range of factors such as caregiver mental and physical health, social involvement, and quality of life (20). To help understand *when* consideration of carer burden in HTAs is more likely to be relevant, the conceptual framework suggested here (Figure 1) is based on the intuitive idea that carer burden is likely to be greatest in conditions that require high-intensity care (e.g., strenuous physical, mental, or emotional effort by caregivers) for an extended period (e.g., years rather than days or weeks). New treatments or modes of delivery for such conditions are likely to have the most potential to reduce caregiver burden and therefore the greatest justification for investing in collecting and analyzing high-quality data on caregiver burden to inform their holistic value.

Duration here refers to the length of time care is required (i.e., during an adaptation period for the patient vs. long-term or chronic conditions) for the care recipient. In contrast, defining the intensity of care is more complex, as it depends on individual circumstances that the caregiver faces, including the needs (i.e., health status and severity of disease) and relationship proximity of the care recipient, the caregiver’s characteristics (e.g., age and gender), psychological and physical health (21), the support they may receive (22), and economic factors (e.g., employment status).

In this literature, measures of caregiver intensity range from the number of hours spent providing care (23–25), the number of caregiving tasks performed (26), to consideration of more complex care areas that can include behavior supervision, complex nursing care, pain management, managing other homecare workers, and advocacy in navigating the healthcare system. In the context of this work, we define caregiving intensity as the time spent caregiving (e.g., number of hours per day), where an increase in time related to caregiving can be associated with high or increasing care needs, indicating the progression of disease and/or decreased functional status of the care recipient, which can all be considered substantial impacts on caregiver burden (23). Therefore, the intensity of caregiving is measured by time inputs, but the valuation of intensity may be complex and include wider concepts such as those affecting caregiver well-being (i.e., impacting life fulfillment/purpose/satisfaction beyond direct HRQoL) (27). This may include additional factors that contribute to the “mental burden” of caregiving beyond capturing the true physical and emotional burdens beyond time commitments. This could be determined by the impact on the caregiver’s life where they have to reorganize their own, existing routines and commitments around required caregiving activities that result in reduced time available for other activities such as employment, volunteering, or leisure/social activities (18).

To capture the impact of caregiving appropriately, we suggest a framework divided into four quadrants. Each quadrant is linked to a high/some/low relevance judgment, which can provide some guidance around the relevance of the caregiver perspective (28). The “high relevance” quadrant identifies disease areas where caregiver burden is highest. Conditions in this quadrant have the greatest absolute potential for (new) treatments to reduce carer burden. Thus, the justification for decision makers to consider high-quality caregiver data collected by the sponsor on the impact of treatment on caregiver burden and its subsequent impact on the health system and benefits beyond the patient is more relevant. This quadrant could include those diseases that are considered intense and often lifetime, chronic conditions, for example, childhood neurological conditions, dementia, muscular dystrophies, and so on, where including the caregiver perspective would provide a more comprehensive assessment of treatment benefits and would therefore require the development of suitable, robust methods and guidance to evaluate all relevant treatment benefits. Other diseases could fall into the “some relevance” category that may represent disease areas with either high intensity of care, that is, cancer, or less intense long-term chronic conditions such as diabetes or cystic fibrosis. These categories may impact individuals beyond the caregiver’s well-being and have potentially larger bereavement effects. The “low relevance” category includes transitional diseases, for example, respiratory illness where the caregiving intensity is low and caregiving activities are required for a limited time period.

We believe this framework establishes a common frame of reference around caregiver burden and can function as a “conversation starter” for researchers, health technology developers, decision makers, and caregivers. It does not, however, aim to narrowly define each quadrant, which may depend on country-specific regulations and available guidance. It would therefore require an individual approach from each agency, similar to how reimbursement agencies assign a “severity premium” for health gains in more severe health states. Although some agencies prefer an explicit consideration of value, including the increase in the acceptable cost of a QALY (e.g., Commission for Pharmaceutical Care (CFH) in the Netherlands), adjustment of the threshold value (e.g., NIPH in Norway), and applying “severity modifiers” (e.g., NICE in England), other agencies prefer to address the issue on a case-by-case basis (i.e., TLV Sweden) (28). Nevertheless, this conceptual framework can help guide policy when identifying the relevance of the caregiver burden (28). Notwithstanding these minor differences in approach, we believe this straightforward framework could provide practical and objective guidance on the relevance of the caregiver perspective in different HTA submissions.

A slight complication arises, however, from the dynamic nature of caregiving intensity and duration, as the characteristics of a particular condition may move from quadrant to quadrant depending on patient needs and treatments. For example, early-stage Alzheimer’s disease (AD) can start with limited impact on caregivers, with low-intensity caregiving required, which may place it in the “low relevance” quadrant of the framework. However, with the progression of the disease, the impact on caregiver burden can move from “low relevance” to “some relevance,” until the caregiver burden is categorized as “highly relevant” in later stages of the disease, characterized by a patient’s complete reliance on the caregiver. Thus, a requirement is for decision makers and health technology developers to continually reassess the appropriateness of including the caregiver perspective in their assessments given changes in disease progression or impact of treatment(s) that may impact reimbursement decisions.

A second complication in interpreting the framework is that a treatment or intervention can have differing, or even offsetting, effects for patients and caregivers, based on the health technology, the (stage of) illness, and the type of intervention, including mode of administration and frequency. For example, the “carer QALY trap” describes a “net negative” scenario where treatments that extend patient survival but with no or minimal improvements in QoL can have the effect of extending caring *duration* with no offsetting reduction in carer *burden (29).* In this scenario, the QALY impact of an extended QoL decrement on the carer can reduce or even outweigh survival gains to the patient, resulting in a net loss of QALYs relative to a “no treatment” scenario. This shows that simply targeting conditions that fall into the higher carer burden quadrant may not always maximize expected QALY gains. Therefore, our proposed framework should not be interpreted as a tool for identifying potential QALY gains, but rather as a guide for understanding when consideration of carer burden is more or less likely to influence the final HTA decision. As some therapies will extend survival or prolong the current health status of patients, the burden on caregiving in these patient groups will likely increase.

## Additional considerations – beyond the “when”

Additional factors beyond the scope of this work that HTA agencies need to consider include defining who qualifies as a caregiver, addressing the impact of bereavement, and how to accurately measure HRQoL. A key question pertains to the definition of caregiving, that is, is it someone who cares for a patient (caregiver impacts) or who cares about a patient (family or network effect), or both (30;31)? Furthermore, a greater understanding of the dynamics of bereavement, including how the effect differs by the circumstances of the patient and the carer before it can realistically be incorporated into HTA, ensuring that bereavement is reflective of the caregiver’s perspective, and not a “utility improving event” as seen in previous technology appraisals (32).

Additional considerations include how to accurately measure the differentiation between decrements in caregivers’ HRQoL that are associated with caregiving compared to those associated with the general impact of aging or health changes (e.g., chronic conditions and comorbidities) in the caregiver (33). Furthermore, the consequences on commonly used cost-effectiveness thresholds as well as implications for reimbursement decisions need to be explicitly communicated by agencies when considering the inclusion of a caregiver perspective (19). We also recognize that consideration of carer HRQoL alongside patient HRQoL could have implications for how to “jointly” consider patient and caregiver severity. Severity modifiers assign greater value to health gains to individuals in more severe health states, often measured in terms of absolute or proportional health shortfalls relative to a disease-free state. Currently, it is unlikely that the health shortfalls associated with caring would ever reach the levels required for eligibility under existing severity modifiers (28), but further research investigating societal preferences, including the appropriate thresholds for “severe” shortfalls associated with caring, and the relative value of carer versus patient health shortfalls and QALY gains, is needed.

## Conclusion

To ensure that HTA decisions are appropriately aligned with health system aims, HTA agencies must provide clearer rationale and guidance for when and why the carer perspective is relevant to HTA decision making. Herein, we have described a framework that could provide guidance objectively and transparently that could reassure developers that investments in collecting high-quality evidence around the impact of new technologies on caregivers alongside patients would be justified by its consideration as part of HTA processes.

Although challenges remain, the clear guidance provided by our proposed framework represents a key step toward ensuring that the carer perspective is appropriately considered by HTA agencies.
